# Increased *O*-GlcNAcylation promotes IGF-1 receptor/PhosphatidyI Inositol-3 kinase/Akt pathway in cervical cancer cells

**DOI:** 10.1038/s41598-022-08445-0

**Published:** 2022-03-16

**Authors:** Victoria Jiménez-Castillo, Daniela Illescas-Barbosa, Edgar Zenteno, Beatriz Xóchitl Ávila-Curiel, Maria Cristina Castañeda-Patlán, Martha Robles-Flores, Daniel Montante-Montes De Oca, Eduardo Pérez-Campos, Anayetzin Torres-Rivera, Abdelouhab Bouaboud, Patrick Pagesy, Carlos Josué Solórzano-Mata, Tarik Issad

**Affiliations:** 1 National Technology of Mexico/IT.Oaxaca, Oaxaca, Mexico; 2 Faculty of Medicine and Surgery, Universidad Autónoma Benito Juárez de Oaxaca, Oaxaca, Mexico; 3 Faculty of Dentistry, Universidad Autónoma Benito Juárez de Oaxaca, Oaxaca, Mexico; 4 Departamento de Bioquímica, Facultad de Medicina, Universidad Nacional Autónoma de México (UNAM), Mexico City, Mexico; 5 Instituto Nacional de Ciencias Médicas y Nutrición Salvador Zubirán, Mexico City, Mexico; 6 Université Paris Cité, Institut Cochin, INSERM, CNRS, 75014 Paris, France; 7 Tecnológico de Estudios Superiores de Huixquilucan, Magdalena Chichicaspa, Mexico

**Keywords:** Nutrient signalling, Cervical cancer, Hormone receptors

## Abstract

*O*-linked β-N-acetylglucosaminylation (*O*-GlcNAcylation) is a reversible post-translational modification on serine and threonine residues of cytosolic, nuclear and mitochondrial proteins. O-GlcNAcylation level is regulated by OGT (O-GlcNAc transferase), which adds GlcNAc on proteins, and OGA (O-GlcNAcase), which removes it. Abnormal level of protein *O*-GlcNAcylation has been observed in numerous cancer cell types, including cervical cancer cells. In the present study, we have evaluated the effect of increasing protein *O*-GlcNAcylation on cervical cancer-derived CaSki cells. We observed that pharmacological enhancement of protein *O*-GlcNAcylation by Thiamet G (an inhibitor of OGA) and glucosamine (which provides UDP-GlcNAc substrate to OGT) increases CaSki cells proliferation, migration and survival. Moreover, we showed that increased *O*-GlcNAcylation promotes IGF-1 receptor (IGF1R) autophosphorylation, possibly through inhibition of protein tyrosine-phosphatase 1B activity. This was associated with increased IGF-1-induced phosphatidyl-Inositol 3-phosphate production at the plasma membrane and increased Akt activation in CaSki cells. Finally, we showed that protein *O*-GlcNAcylation and Akt phosphorylation levels were higher in human cervical cancer samples compared to healthy cervix tissues, and a highly positive correlation was observed between *O*-GlcNAcylation level and Akt phosphorylation in theses tissues. Together, our results indicate that increased *O*-GlcNAcylation, by activating IGF1R/ Phosphatidyl inositol 3-Kinase (PI-3K)/Akt signaling, may participate in cervical cancer cell growth and proliferation.

## Introduction

**Figure 1:**
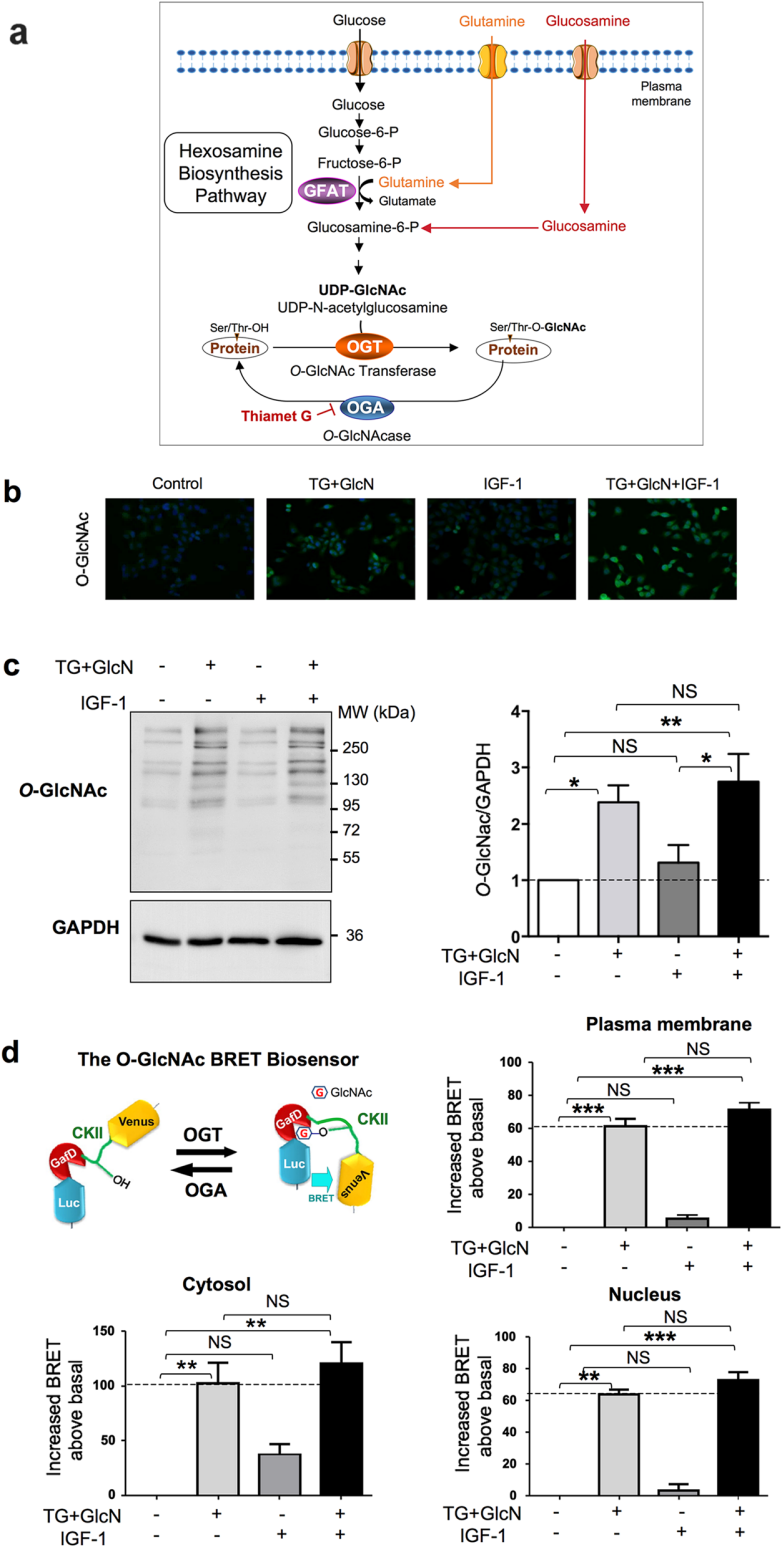
Effect of Thiamet-G and glucosamine on *O*-GlcNAcylation levels in CaSki cells. (**a**) Glucose and glutamine metabolism in the hexosamine biosynthesis pathway (HBP) leads to the production of UDP-GlcNAc (Uridine 5-diphospho N-acetylglucosamine), the substrate used by OGT for *O*-GlcNAcylation of cytosolic, nuclear and mitochondrial proteins. The rate-limiting step of the HBP is catalysed by GFAT (glutamine fructose-6-phosphate amidotransferase) which uses glutamine to convert fructose-6 phosphate into glucosamine-6 phosphate. Experimentally, the level of *O*-GlcNAcylation of proteins can be increased by incubating cells with glucosamine (which bypasses the GFAT rate-limiting enzyme), or with Thiamet G, which inhibits deglycosylation of proteins by OGA. (**b**) CaSki cells were grown in 1% FBS and cultured in the absence or presence of TG (10 µM for 24 h) and GlcN (5 mM for 6 h) and then stimulated with IGF-1 (5 nM for 10 min). Cells were fixed and immunofluorescence imaging was performed using anti-*O-*GlcNAc antibody (green). DAPI (blue) was used to visualize the nucleus. Representative images are shown. (**c**) Cells were cultured in 1% FBS and cultured in the absence or presence of TG (10 µM) for 24 h, GlcN (5 mM) for 6 h and IGF-1 (5 nM) for 10 min. Cell lysates were collected and analysed by western blot with anti-*O*-GlcNAc antibody (the uncropped western-blots are shown on the Supplementary Information file S1). The histogram represents the means ± SEM of 8 independent experiments. (**d**) The BRET *O*-GlcNAc biosensor is composed of Rluc8 luciferase fused to a lectin domain (GafD), followed by a known OGT substrate peptide derived from casein kinase II (CKII), and then by the Venus variant of the yellow fluorescent protein. Upon its *O*-GlcNAcylation, the casein kinase peptide binds to the lectin, resulting into a conformational change detected as an increased BRET signal. Cells were transfected with cDNAs coding for cytosol-, nucleus- and plasma membrane-targeted BRET *O*-GlcNAc-biosensors. Results are expressed in milliBRET units (mBU) as increased BRET above basal induced by the different agents, and are the means ± SEM of 6 independent experiments. Statistical analysis was performed using ANOVA followed by Tukey’s post-test. *, **, ***p < 0.05, p < 0.01 and p < 0.001, respectively. *NS* non-significant.

*O*-linked β-N-acetylglucosaminylation (*O*-GlcNAcylation) is a reversible post-translational modification (PTM) that occurs on cytosolic, nuclear, and mitochondrial proteins. It consists in the addition of N-Acetyl glucosamine (GlcNAc) on serine and threonine residues. Only two enzymes control the *O*-GlcNAc level on proteins: OGT (*O*-GlcNAc transferase), which adds the GlcNAc motif on proteins, and OGA (*O*-GlcNAcase), which removes it^1^ (Fig. 1a).

Alike phosphorylation, O-GlcNAcylation regulates the activity, the stability, and the subcellular localization of proteins. More than 4000 different O-GlcNAcylated proteins have been identified so far. O-GlcNAcylation can compete directly with phosphorylation at the same sites or regulate phosphorylation on adjacent sites, providing an intricate crosstalk between the two PTM in order to control a number of protein functions and biological processes. Perturbations in protein O-GlcNAcylation have been involved in various diseases, including neurodenerative and cardiovascular diseases, diabetes and cancer. Interestingly, UDP-GlcNAc, the substrate used by OGT to O-GlcNAcylate proteins (Fig. 1a), is produced in the hexosamine biosynthetic pathway from glucose and glutamine, two obligatory nutrients for cancer cells^2^. *O*-GlcNAcylation can promote tumor development by perturbation of the activity of signaling pathways, oncogenic factors and epigenetic regulators^2^. Increased OGT expression and protein *O*-GlcNAcylation have been observed in numerous cancer cells, including gastric, breast, lung, prostate, colon, liver, bladder, ovarian and endometrial cancer cells^2^.

More recent studies have also revealed the involvement of O-GlcNAcylation in cervical cancer^3–7^. Cervical cancer is fourth most common female malignancy worldwide, with half a million women diagnosed each year^8^. The most important etiological factor is the infection with the human papilloma virus (HPV)^8^. The E6/E7 oncogenes encoded by the high-risk HPV-16 and HPV-18 were shown to increase OGT levels and protein O-GlcNAcylation, and promote cell proliferation in mouse embryonic fibroblasts^5^. Moreover, pharmacological or genetic inhibition of OGT in HPV-transformed cervical carcinoma cells impaired the transformed phenotype^5^. O-GlcNAcylation has been shown to promote cervical cancer cell proliferation, tumorigenesis and metastasis through various mechanisms, including increased cMyc stability^5^, NFκB-mediated CXCR4 (C-X-C Motif Chemokine Receptor 4) expression^6,7^, LXR (Liver X Receptor)-induced clusterin expression^9^ as well as HCF-1 (Host Cell Factor-1) and MLL (Mixed Lineage Leukemia) 5-mediated increase in E6/E7 expression^3,4^.

The IGF-1R/PI-3K/Akt (IGF-1 receptor/Phosphatidyl Inositol-3 kinase/Akt) axis is considered an essential target for cervical cancer treatment^10–14^. Several lines of evidence have suggested that *O*-GlcNAcylation may affect cancer cell proliferation/apoptosis by activating the PI-3K/Akt pathway^15–18^. However, the effect of *O*-GlcNAcylation on PI-3K/Akt pathway has not been explored in cervical cancer cells. In this work, we evaluated the consequences of increased *O*-GlcNAcylation on IGF-1 effects in cervical-cancer-derived CaSki cells. We observed that *O*-GlcNAcylation-inducing agents promoted IGF1-induced proliferation and migration. Moreover, we showed that increased *O*-GlcNAcylation was associated with increased IGF1R activation, Phosphatidyl Inositol-3 Phosphate (PIP_3_) production, and Akt phosphorylation. Finally, we observed a positive correlation between protein *O*-GlcNAcylation and Akt phosphorylation levels in cervical tumor samples, confirming the link between these two pathways in human cervical cancer.

## Results

### Effect of *O*-GlcNAcylation-inducing agents on CaSki cells

*O*-GlcNAcylation-inducing agents promote cell proliferation and migration in various cancer cells. To investigate the effect of *O*-GlcNAcylation on IGF-1-induced proliferation and migration of cervical uterine cancer-derived cells, CaSki cells grown in medium containing 1% serum were treated with Thiamet-G (a selective OGA inhibitor) and glucosamine (which bypasses the rate-limiting step of the hexosamine biosynthesis pathway **(**HBP, Fig. 1a)), in absence or presence of IGF-1. As shown in Fig. 1b,c, combined treatment with Thiamet G plus glucosamine (TG + GlcN) markedly increases protein *O*-GlcNAcylation, as detected using an anti-*O*-GlcNAc antibody by immunofluorescence (Fig. 1b) and by western-blot (Fig. 1c). In the presence of IGF-1, a tendency toward an increase in protein *O*-GlcNAcylation was observed, but this increase was not statistically significant, neither in the absence or presence of TG + GlcN (Fig. 1c). Subcellular relocalization of OGT in different cell compartments has been observed upon stimulation of tyrosine kinase receptors^19,20^, resulting in compartment-specific changes in *O*-GlcNAcylation. To evaluate whether IGF-1 treatment may affect protein *O*-GlcNAcylation in different compartments in CasKi cells, we used a BRET-based *O*-GlcNAc-biosensor comprising a lectin domain and a Casein Kinase II-derived *O*-GlcNAcylation site (Fig. 1d). Increased *O*-GlcNAcylation induces a conformational change in this biosensor, resulting in an increased BRET signal^21^. Using different versions of the *O*-GlcNAc-biosensor targeted to the plasma membrane, the cytosol, or the nucleus^22^, we evaluated *O*-GlcNAcylation activity in CasKi cells treated with TG + GlcN and stimulated or not with IGF-1 (Fig. 1d). TG + GlcN treatment markedly increased BRET signal in all three compartments. Upon IGF-1 treatment, a tendency toward an increase in BRET signal was observed in the cytosol, but this effect did not reach statistical significance. IGF-1 had no effect on BRET in the nucleus or plasma membrane. Together, these results show that TG + GlcN treatment strongly stimulates protein *O*-GlcNAcylation in CasKi cells, whereas IGF-1 has no significant impact on *O*-GlcNAcylation level.

**Figure 2:**
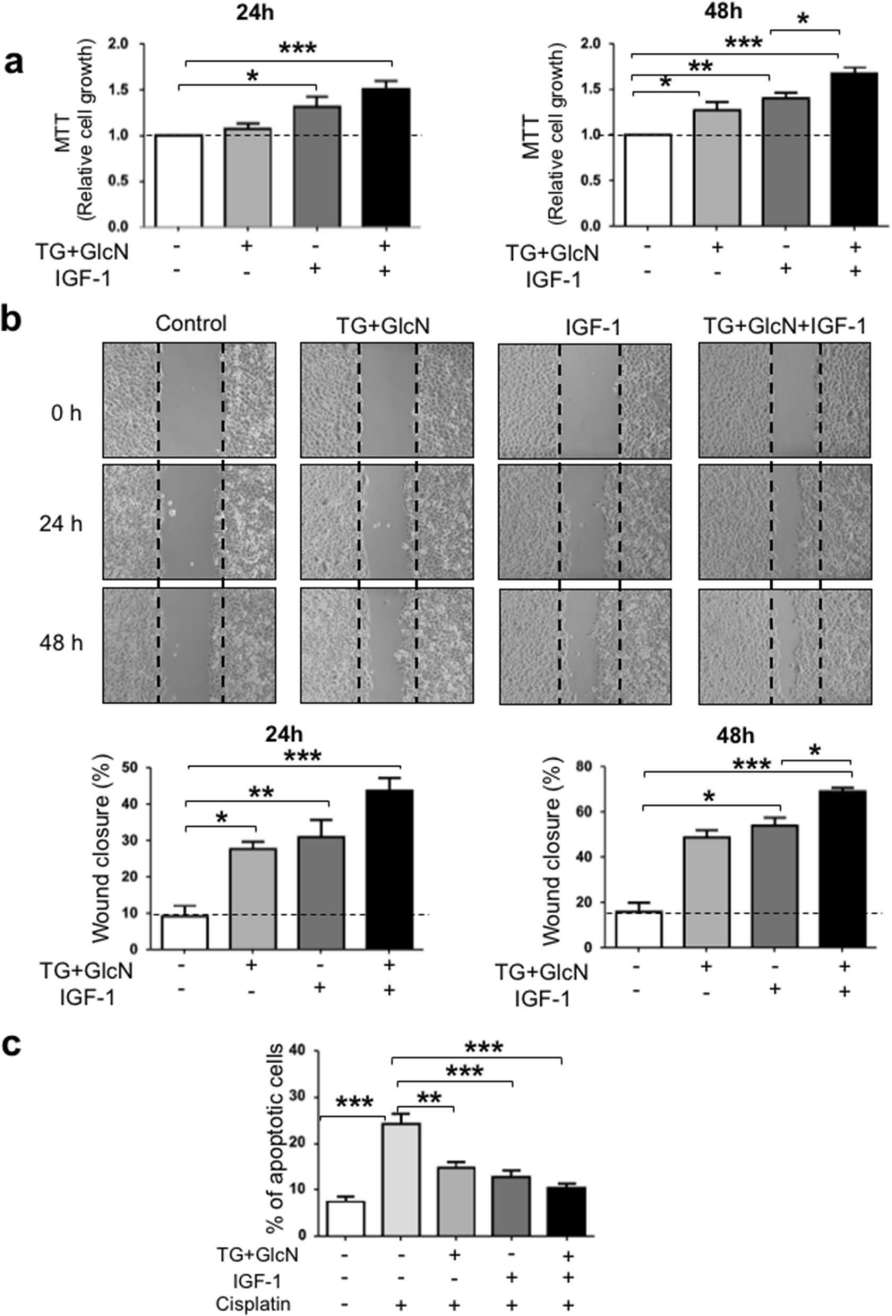
*O*-GlcNAcylation-inducing treatments increase cell growth and migration and inhibit apoptosis in CaSki cells. (**a**) Cells were cultured in 1% FBS and cultured in the absence and presence of *O*-GlcNAcylation-inducing agents (TG 10 µM and GlcN 5 mM) and IGF-1 (5 nM). MTT assay was used to determine the cell growth at 24 h and 48 h. Results are the mean ± SEM of 5 independent experiments. (**b**) Wound healing assay was performed as described in “Methods”. Representative images of the wound at 0, 24 and 48 h are shown. Migration is expressed as the wound closure percentage at 24 h and 48 h. The results are presented as mean ± SEM of 4 independent experiments. (**c**) CaSki cells were treated for 24 h with *O*-GlcNAcylation-inducing agents, IGF-1 and Cisplatin, and then stained with Annexin V and propidium iodide for FACS analysis. The results are presented as mean ± SEM of 4 independent experiments. Statistical analysis was performed using ANOVA followed by Tukey’s post-test. *, **, ***p < 0.05, p < 0.01 and p < 0.001, respectively.

We then evaluated the effect of these treatments on cell growth, migration and apoptosis. As shown in Fig. 2a, after 24 h of culture, the growth of CaSki cells was not significantly affected by TG + GlcN treatment, whereas it was significantly increased by IGF-1 treatment. However, after 48 h, treatment with TG + GlcN significantly increased cell growth compared to untreated cells. Moreover, in the presence of TG + GlcN, the effect of IGF-1 was significantly increased when compared to the effect of IGF-1 alone.

To investigate the effects of *O-*GlcNAcylation on cell migration, CaSki cells were submitted to a wound-healing assay (Fig. 2b). A linear wound in a confluent cell monolayer was made, and images were taken at different healing times in the absence or presence of *O*-GlcNAcylation-inducing agents and/or IGF-1. After 24 h-treatment, we observed that TG + GlcN favored wound closure both in the absence and presence of IGF-1. This effect was more pronounced after 48 h-treatment, showing significant improvement in wound healing upon treatment with both IGF-1 and TG + GlcN, when compared to IGF-1 alone.

Protein *O*-GlcNAcylation can promote cell proliferation, but has also been involved in sensitivity to apoptosis induced by anticancer drugs in breast^16,23^, gastric^24^, lung^25^ and colon-derived cancer cells^26^. We therefore evaluated the effects of *O*-GlcNAcylation-inducing agents (TG + GlcN) on cisplatin-induced cell apoptosis by FACS analysis after labeling the cells with Annexin V-FITC/propidium iodide. We found that a 24 h-treatment with TG + GlcN promoted a decrease in cisplatin-induced apoptosis similar to that obtained with IGF-1 (Fig. 2c). A modest further reduction was observed when cells were treated with both IGF-1 and TG + GlcN compared to IGF-1 alone, but this effect did not reach statistical significance.

Altogether, these experiments indicate that *O*-GlcNAcylation-inducing treatments promote cell growth, migration, and resistance to cell death induced by a chemotherapeutic agent. Moreover, *O*-GlcNAcylation-inducing treatments appear to enhance the effect of IGF-1 on cell proliferation and migration.

### Effect of *O*-GlcNAcylation-inducing treatments on IGF-1R phosphorylation

**Figure 3:**
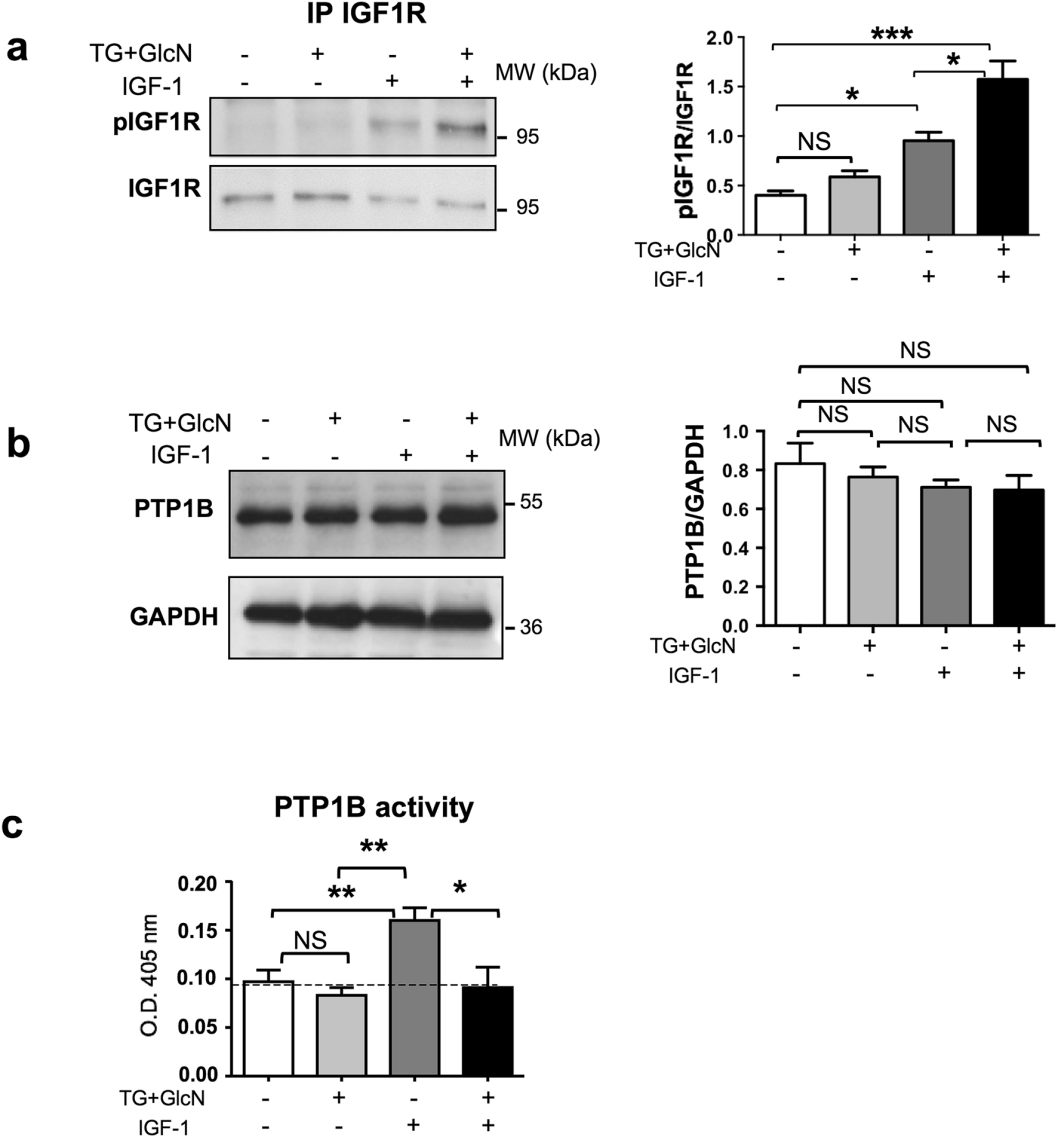
Effect of *O*-GlcNAcylation-inducing treatments on IGF-1R phosphorylation and PTP1B expression and activity in CaSki cells. CaSki cells were cultured in the presence of 1% FBS in the absence or presence of TG (10 µM for 24 h) and GlcN (5 mM for 6 h). Cells were then stimulated with IGF-1 (5 nM) during 10 min. (**a**) CaSki cells were lysed and IGF1R were immunoprecipitated with an anti-IGF1R antibody. IGF1R phosphorylation was analysed by western blotting using an anti-phospho-IGF1R antibody. The blots were reprobed with an anti-IGF1R antibody (the uncropped western-blots are shown on the Supplementary Information file S1). The histogram represents the ratio of P-IGF1R/IGF1R signal obtained by densitometric analysis of the blots. Results are the means ± SEM of 3 independent experiments. (**b**) CaSki cells were cultured in presence of 1% FBS in the absence or presence of TG (10 µM for 24 h) and GlcN (5 mM for 6 h). Cells were then stimulated with IGF-1 (5 nM) during 10 min. Cells were lysed and PTP1B expression was evaluated by western-blotting (the uncropped western-blots are shown on the Supplementary Information file S1). The histogram represents the ratio of PTP1B/GAPDH signal obtained by densitometric analysis of the blots. Results are the means ± SEM of 4 independent experiments. (**c**) Cell lysates were immunoprecipitated using an anti-PTP1B antibody and PTP1B activity was measured using p-nitrophenyl-phosphate (pNPP) as a substrate. Histograms represent the means ± SEM of PTP1B enzymatic activity (optical density at 405 nm) in 4 independent experiments. Statistical analysis was performed using ANOVA followed by Tukey’s post-test. *; **; ***p < 0.05, p < 0.01, and p < 0.001, respectively. *NS* non-significant.

Since *O*-GlcNAcylation appears to enhance IGF-1 effect on cell proliferation and migration, we evaluated the effect of *O*-GlcNAcylation-inducing treatments on IGF-1 signaling. IGF-1 induces its biological effects on cells through activation of a transmembrane tyrosine kinase receptor (IGF1R). Binding of IGF-1 induces autophosphorylation of the IGF1R on tyrosine residues. More specifically, full activation of the receptor by its ligands requires the autophosphoryation of the three tyrosines located in the kinase domain^27^. To determine whether *O*-GlcNAcylation may improve IGF-1 effects on Caski cells by promoting the activation of IGF1R, cells were cultured in the absence or presence of TG + GlcN and then stimulated for 5 min. with IGF-1. IGF1R was then immunoprecipitated with an anti-IGF1R antibody and probed with an anti-phospho-IGF1R antibody directed against the tris-phophorylated form of the kinase domain (Fig. 3a). We observed that whereas TG + GlcN did not affect basal IGF1R phosphorylation, it significantly increased IGF-1-induced phosphorylation of the three tyrosines of the kinase domain.

We reasoned that increased tyrosine phosphorylation of the IGF1R by *O*-GlcNAcylation-inducing treatments could be the result of a decrease in the expression and/or activity of the protein tyrosine phosphatases (PTPases) that regulate the receptor. PTP1B is one of the main PTPases that control IGF1R phosphorylation^28^. We observed that PTP1B expression was not significantly modified by TG + GlcN treatment (Fig. 3b). To determine whether *O*-GlcNAcylation inducing treatment may have affected PTP1B activity, PTP1B was immunoprecipitated, and its activity was measured using p-nitrophenyl-phosphate as a substrate (Fig. 3c). As described previously, IGF-1 treatment significantly increased PTP1B enzymatic activity^29^. Interestingly, TG + GlcN treatment had no significant effect on basal PTP1B activity but markedly impaired the stimulatory effect of IGF-1 on the activity of the enzyme. These results suggest that *O*-GlcNAcylation-inducing treatments may promote IGF1R signaling by reducing feed-back inactivation of IGF1R by PTP1B.

### *O*-GlcNAcylation-inducing treatments increase IGF-1-stimulated PIP_3_ production in CaSki cells

**Figure 4:**
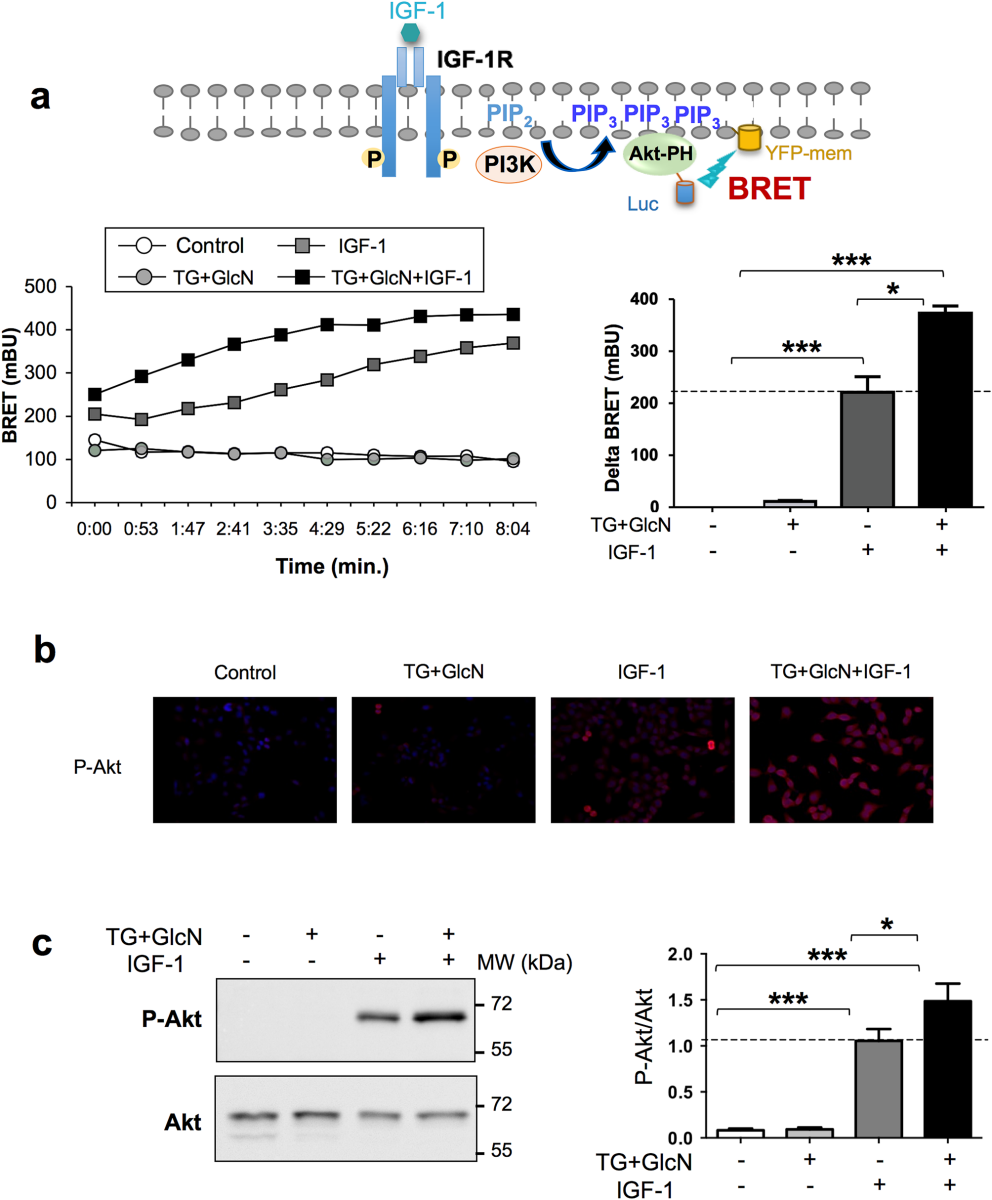
*O*-GlcNAcylation-inducing treatments increase IGF-1 effects on PI-3 kinase/Akt pathway in CaSki cells. (**a**) Activation of PI-3 kinase induces the phosphorylation of phosphatidyl-inositol 2 phosphate (PIP_2_) into phosphatidyl-inositol 3 phosphate (PIP_3_) and subsequent recruitment of Akt to the plasma membrane through its pleckstrin homology (PH) domain. CaSki cells were co-transfected with cDNAs coding for the PH domain of Akt fused to a luciferase (Luc-Akt-PH) and a plasma membrane-targeted YFP (YFP-mem). 24 h after transfection, cells were cultured in the presence of 1% FBS in the absence or presence of TG (10 µM for 24 h) and GlcN (5 mM for 6 h). Cells were incubated with coelenterazine for 10 min, and then stimulated with IGF-1 (5 nM). Light emission acquisition at 480 nm and 532 nm was started immediately after IGF-1 addition. Results were expressed in miliBRET units (mBU). Left panel: a typical real-time experiment showing the effect of *O*-GlcNAcylation-inducing treatment on IGF-1-induced PIP_3_ production in CaSki cells. Right panel: Results are expressed as the delta BRET (increased BRET above basal) and are the means ± SEM of 6 independent experiments. (**b**) CaSki cells were cultured in the presence of TG (10 µM) for 24 h and GlcN (5 mM) for 6 h, stimulated with IGF-1 for 10 min, and then fixed and incubated with P-Akt antibody. Representative images of immunofluorescence with P-Akt (red) and dapi staining of cell nuclei (blue) are shown. (**c**) CaSki cells were cultured in the presence of TG (10 µM for 24 h) and GlcN (5 mM for 6 h), stimulated with IGF-1 for 10 min, and then lysed. Proteins were submitted to western-blotting and Akt phosphorylation was detected with anti-phospho-Akt antibody (the uncropped western-blots are shown on the Supplementary Information file S1). The histogram represents the ratio of P-Akt/Akt signals obtained by densitometric analysis of the blots. Results are the means ± SEM of 8 independent experiments. Statistical analysis was performed using ANOVA followed by Tukey’s post-test. *, ***p < 0.05, p < 0.001, respectively. *NS* non-significant.

The PI-3 kinase/Akt pathway plays a major role in mediating IGF-1 effects on cell proliferation, migration, and apoptosis. Activation of PI-3 kinase by tyrosine kinase receptors promotes the phosphorylation of phosphatidyl-inositol 2 phosphate (PIP_2_) into phosphatidyl-inositol 3 phosphate (PIP_3_) at the plasma membrane, which then induces the recruitment, phosphorylation, and activation of Akt. In order to determine whether *O*-GlcNAcylation affects this signaling pathway in CaSki cells, we used a BRET-based biosensor that monitors, in real-time, in living cells, PIP_3_ production at the plasma membrane^16,30–32^. CaSki cells were co-transfected with cDNAs coding for the pleckstrin homology (PH) domain of Akt fused to a luciferase (Luc-Akt-PH), and YFP targeted to the plasma membrane (YFP-mem) (Fig. 4a). As shown in Fig. 4b, treatment with *O*-GlcNAcylation-inducing agents had no effect on basal PIP_3_ production, whereas IGF-1 rapidly increases PIP_3_ production. However, the presence of *O*-GlcNAcylation-inducing agents markedly increased the effect of IGF-1 on PIP_3_ production. This result suggests that *O*-GlcNAcylation-inducing agents increase IGF-1 stimulatory effect on PI-3 kinase signaling.

### *O*-GlcNAcylation-inducing treatments increase IGF1-stimulated Akt phosphorylation in CaSki cells

We then evaluated Akt phosphorylation level in CaSki cells by immunofluorescence staining (Fig. 4b) and by western blotting using anti-phospho-Akt antibody (Fig. 4c). We observed that TG + GlcN had no detectable effect on Akt phosphorylation, whereas IGF-1 markedly increased Akt phosphorylation level. In agreement with the effect of TG + GlcN treatment on PIP_3_ production, this treatment significantly increased the effect of IGF-1 on Akt phosphorylation (Fig. 4c). In contrast, these agents did not significantly affect Erk phosphorylation, indicating a selective effect of increased *O*-GlcNAcylation on IGF-1-induced PI-3K/Akt pathway (Suppl. Fig. S1).

Together, these results indicate that increased *O*-GlcNAcylation in CaSki cells promotes IGF-1-induced IGF1R phosphorylation and PI-3 kinase/Akt signaling pathway.

### *O*-GlcNAcylation and Akt phosphorylation levels are elevated in cervical cancer tissues

**Figure 5:**
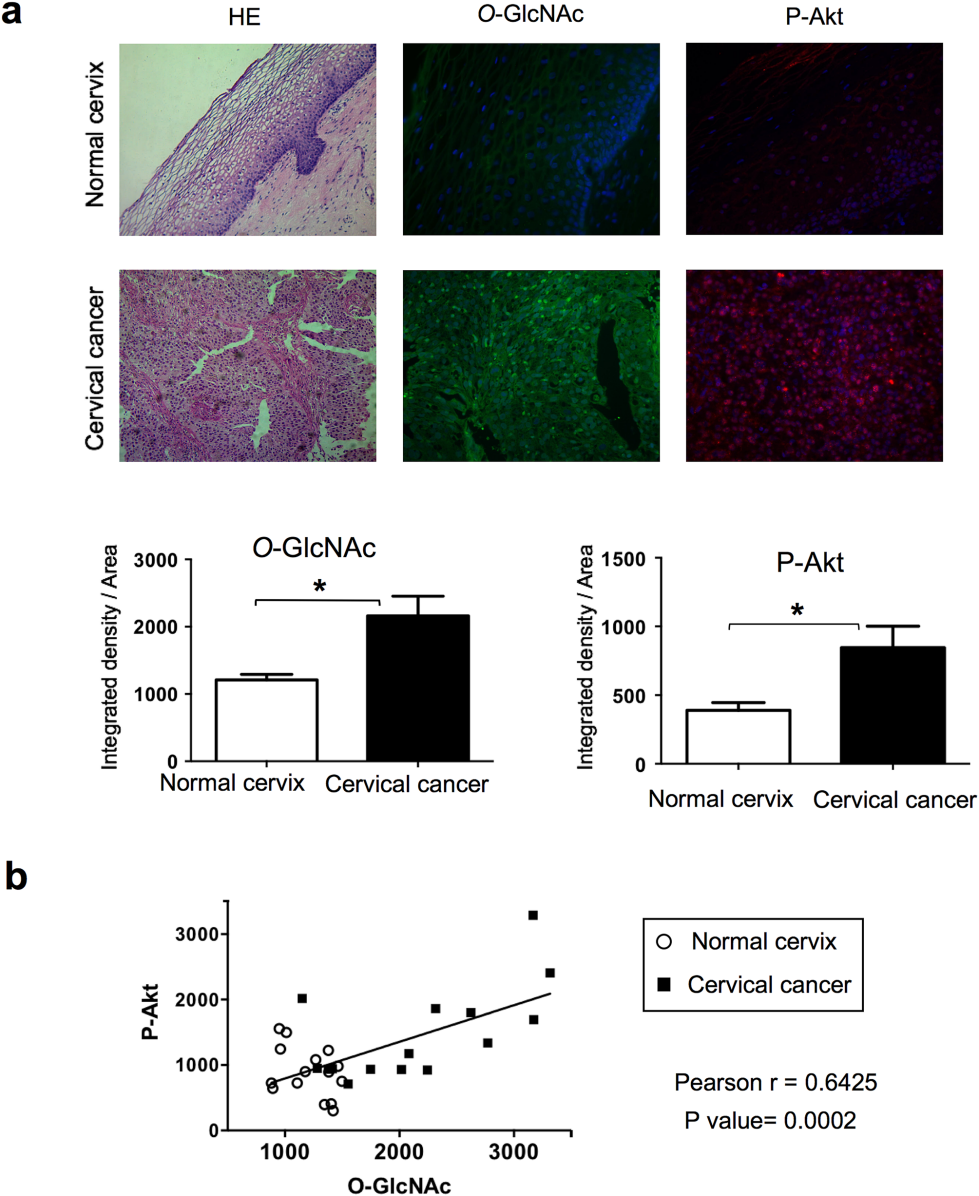
O-GlcNAcylation and Akt phosphorylation levels in cervical cancer tissues. (**a**) Tissues were stained with Hematoxylin/Eosin (HE). Indirect inmunofluorescence was performed using indicated primary antibodies followed by secondary antibodies (FITC-conjugated anti-mouse and biotinylated anti-rabbit/Alexa Fluor 594). Representative merged images of immunofluorescence staining for *O*-GlcNAc (green), P-Akt (red) and cell nuclei (blue) of normal and cervical cancer-tissues are shown. The histograms display the relative quantification of the mean fluorescence integrated density by Image J software. Data are means ± SEM of n = 5 tissues, analysing each tissue in at least 3 different optical fields. Statistical analysis was performed using a Student’s t-test. *p < 0.05. (**b**) Positive correlation between *O*-GlcNAc and phopho-Akt signals in samples from normal and cervical cancer-tissues evaluated by Pearson analysis.

Our results suggest a link between increased *O*-GlcNAcylation and PI-3 kinase/Akt signaling pathway in cervical cancer cells. To determine whether a correlation between *O*-GlcNAcylation and PI-3 kinase/Akt pathway could be observed in human cervical cancer, we evaluated Akt phosphorylation (P-Akt) and protein *O*-GlcNAcylation levels in cervical cancer tissues and healthy cervix by immunofluorescence staining. As shown in Fig. 5a, cervical cancer exhibited significantly higher *O*-GlcNAcylation levels and P-Akt signal intensity compared to normal cervical tissue, in agreement with a link between *O*-GlcNAcylation and increased activity of the PI3-kinase/Akt pathway in cervical cancer. Moreover, linear regression analysis indeed indicated a highly significant (p = 0.0002) positive correlation between *O*-GlcNAc level and Akt phosphorylation in cervical tissues (Fig. 5b).

## Discussion

In humans, excess food intake associated with modern lifestyle constitutes an important cancer risk factor^2^, and cervical cancer has been positively associated with body mass index (BMI) and inversely with physical activity^33^. In mouse and rat models, overfeeding is associated with faster development of tumors^34^. In contrast, food restriction has inhibitory effects on tumor growth in rodents^35^ and reduces cancer incidence in non-human primates^36^. Interestingly, when grown as tumor xenografts in mice, cancer cells with mutations inducing constitutive PI3K activation are resistant to dietary restriction, whereas cancer cells with mutations that constitutively activate the Ras/Raf/MAPK pathway remain sensitive to dietary restriction. These observations strongly suggest that the activity of the PI3K/Akt pathway is an important determinant of the sensitivity of cancer cells to nutritional conditions^37^. O-GlcNAcylation is believed to depend on nutritional conditions, and OGT is considered as an important metabolic sensor. Increased protein O-GlcNAcylation is now largely recognized as a hallmark of cancer^2^. In the present work, we observed that increasing *O*-GlcNAcylation promotes proliferation and invasiveness of cervical cancer cells, associated with activation of IGF1R/PI-3 kinase/Akt signaling. To induce protein O-GlcNAcylation, we used a combination of Thiamet G that inhibits OGA and glucosamine which promotes UDP-GlcNAc synthesis. Indeed, preliminary experiments have indicated that a more robust effect on protein O-GlcNAcylation was obtained when both agents were present together (Suppl. Fig. S2). In agreement with this observation, the effect of Thiamet G alone or glucosamine alone on IGF1 induced cell growth and PIP_3_ production were less pronounced (Suppl. Fig. S2).

However, because glucosamine-induced UDP-GlcNAc synthesis may also affect N-glycan synthesis, we evaluated this possibility in control experiments using different lectins. We observed that glucosamine alone or in combination with TG had no significant effect on binding of Concanavalin A, PHA-L (Phytohemagglutinin-l) and WGA (wheat-germ agglutinin) on intact cells (FACS experiments) and total cell lysate proteins (western-blotting experiments) (Suppl. Fig. S3).

The Insulin-like growth factor system has been largely involved in tumor initiation and progression^38^. IGF-1 and 2 act through IGF1R, a tyrosine kinase receptor that belongs to the insulin receptor (IR) family. Whereas the IR is mainly involved in metabolic regulations, the IGF1R primarily mediates growth activities^39,40^ and has become an attractive target for the treatment of numerous cancer types^41^, including cervical cancer^10,11^. Indeed, several lines of evidence indicate that IGF1R plays an important role in growth and survival of cervical cancer cells. Thus, whereas IGF1R protein was nearly undetectable in normal cervical epithelial cells, IGF1R protein was abundant in cervical cancer cell lines and correlated with the high responsiveness of these cells to IGF-1 in proliferation and invasion assays^42^. Moreover, down-regulation of IGF1R using antisense RNA can reverse the transformed phenotype of human cervical cancer cell lines^43^. In agreement with these data, the 5-year recurrence-free and overall survival was significantly lower in patients with high expression level of IGF1R compared to those with low expression levels in cervical cancer tissues^10^.

The PI-3 kinase/Akt pathway is also a major target for cancer therapy, as it is frequently deregulated in human malignancies^44^, including cervical cancer^45^. Most notably, Akt hyperphosphorylation has been shown in cervical cancer specimens, suggesting over activation of the PI3K/Akt pathway in cervical cancer^46^. Besides mutations of PI3KCA (PI-3 kinase catalytic subunit alpha) that lead to constitutive PI-3K activation, E6 and E7 papilloma virus proteins have been suggested to increase Akt phosphorylation, possibly through PTEN (phosphatase and tensin homolog)^47^ or PP2A (protein phosphatase 2A)^48^ regulation. Therefore, the O-GlcNAcylation-induced up-regulation of the IGF1R/PI-3K/Akt axis observed in our study may also have important pathophysiological significance in the context of cervical cancer. Several studies previously reported that increased O-GlcNAcylation promotes Akt activation in other cancers cell types^49^, including thyroid anaplastic cancer cells^15^, breast cancer cells^16^, gastric cancer cells^17^ and pre-B acute lymphocytic leukemia cells^18^. These effects were associated with O-GlcNAcylation-induced increase in cell proliferation, invasiveness and survival. However, the mechanism by which O-GlcNAc promoted the PI-3 kinase/Akt pathway was not investigated in these studies.

In our work, we found that O-GlcNAcylation-inducing treatments increases IGF-1-stimulated autophosphorylation of the IGF1R. This effect appears to involve the regulation of PTP1B activity by O-GlcNAcylation-inducing treatments. PTP1B is one of the main protein tyrosine-phosphatase involved in the regulation of IR and IGF1R signaling^28^. This tyrosine phosphatase has been involved in both metabolic and oncogenic diseases^50^. PTP1B is located on the cytosolic side of the endoplasmic reticulum. It interacts with and dephosphorylates IR and IGF1R after internalization induced by their respective ligands^51,52^. Regulation of IGF1R kinase activity by PTP1B was demonstrated in PTP1B deficient fibroblasts, which display enhanced IGF-1-mediated suppression of apoptosis and motility^53^. In another study, decreased PTP1B expression was observed in various ovarian cancer cell lines compared to normal epithelial cells, and stable restoration of PTP1B was shown to antagonize IGF1R signalling pathway in these cancer cells^54^. We therefore hypothesized that PTP1B could also play an important role in the regulation of IGF1R signaling in cervical cancer cells. We observed that IGF-1 stimulation significantly increased PTP1B activity in these cells (Fig. 3c). Ligand-induced increase in PTP1B activity has been described previously upon stimulation of different tyrosine kinase receptors, including IGF1R and IR^29,55^, Epidermal Growth Factor receptor^56^ and Erythorpoietin receptor^57^, and was interpreted as a feed-back mechanism that down-regulates the tyrosine kinase activity of these receptors. We observed that in cervical cancer cells, this down-regulation was impaired by TG + GlcN, resulting in an increased IGF1R receptor activation (Fig. 3a) and signaling through the PI-3 kinase/Akt pathway (Fig. 4). In contrast, no significant change of Erk activation could be detected under TG + GlcN treatment (Suppl. Fig. S1). Interestingly, differential effects of PTP1B on the activities of PI-3K/Akt and Erk signaling pathways have also been reported previously^58–60^.

The mechanism by which O-GlcNAc-inducing treatment inhibits IGF-1-induced PTP1B activity is unclear at the present time. A previous study showed that in liver cells, PTP1B could be O-GlcNAcylated by high glucose and/or by inhibition of OGA with PUGNAc, associated with decreased insulin-induced Akt phosphorylation^61^. However, we have evaluated the effect of TG + GlcN treatment on PTP1B O-GlcNAcylation and observed that no O-GlcNAc signal above background could be detected using RL2 antibody (Suppl. Fig. S4B). Moreover, because PTP1B activity has been shown to be regulated through interaction with and phosphorylation by tyrosine-kinase receptors, we also evaluated whether TG + GlcN treatment modulated IGF1R/PTP1B interaction. Using BRET technique^52^, we observed that this treatment did not significantly affected basal or IGF-1-induced IGF1R/PTP1B interaction (Suppl. Fig. S4A). Moreover, we did not observe any significant effect of TG + GlcN treatments on phosphotyrosine level on PTP1B (Suppl. Fig. S4C). However, we cannot exclude that subtle changes in the phosphorylation of specific tyrosine residues may remain undetected in these experiments. Indeed, at least 3 regulatory phosphorylated tyrosines (Y66, Y152 and Y153) have been described on PTP1B^56,62^. Clearly, site-directed mutagenesis of each of these tyrosines will be necessary to further explore these mechanisms.

Most importantly, the link between O-GlcNAcylation and PI-3K/Akt pathway in cervical cancer was further indicated by immunofluorescence analysis of Akt phosphorylation and protein O-GlcNAcylation in human samples. Indeed, protein O-GlcNAcylation and Akt phosphorylation levels were higher in cervical cancer samples compared to healthy cervix tissues (Fig. 5a), and a highly significant correlation was observed between protein O-GlcNAcylation and Akt phosphorylation signals (Fig. 5b). Together with our data showing that increased O-GlcNAcylation promotes the PI-3 kinase/Akt pathway, this result strongly supports a crucial role for O-GlcNAcylation in cervical cancer.

In summary, in this work, we showed that O-GlcNAcylation promotes proliferation, migration, and survival of cervical cancer cells. Moreover, we showed that O-GlcNAcylation increases the activity of the IGF1R/PI-3K/Akt signaling pathway in these cells. This suggests that lifestyle and/or therapeutic intervention to limit protein O-GlcNAcylation may constitute interesting approaches for the prevention or treatment of cervical cancer.

## Methods

All methods were carried out in accordance with relevant guidelines and regulations.

### Chemicals and antibodies

Glucosamine (GlcN), Thiamet G (TG), Thiazolyl Blue Tetrazolium Bromide (MTT), Cisplatin, pNPP (4-Nitrophenyl phosphate di(2-amino-2-ethyl-1, 3-propanediol)), and recombinant human IGF-1 were from Sigma-Aldrich. Anti-phospho-Akt, anti-phospho-Erk, anti-IGF-1R and anti-PTP1B antibodies were from Cell Signaling Technology. Anti-Akt, anti-Erk, anti-GAPDH, and HRP-conjugated anti-rabbit antibodies were from Santa Cruz Biotechnology. Anti phospho-IGF-1R antibody (anti-phospho-IR/IGF1R pTyr^1158/1162–1163^) was from Sigma-Aldrich. Immunoprecipitation of PTP1B for enzymatic assays was performed using anti-PTP1B antibody from Calbiochem. Anti-*O*-GlcNAc antibody (RL2) and Streptavidin Alexa Fluor 594 conjugate were from Thermo Fisher Scientific. HRP-conjugated anti-mouse, Fluorescein (FITC)-AffiniPure Goat anti-Mouse, and Biotin-SP-Conjugated AffiniPure Goat anti-Rabbit were from Jackson ImmunoResearch Laboratories.

### Cell culture and transfection

CaSki cells (ATCC CRL-1550) were maintained in RPMI-1640 medium supplemented with 10% fetal bovine serum (FBS) and 1% penicillin–streptomycin at 37 °C in 5% CO_2_ (Life Technologies). cDNA transfections were performed with Lipofectamine 2000 reagent (Life Technologies). For the study of *O*-GlcNAcylation in different cell compartments by BRET, 3.6 × 10^5^ cells/well were transfected with 0.5 µg cDNAs coding for cytosol-, nucleus- or plasma membrane-targeted BRET *O*-GlcNAc-biosensors^22^. For the study of PIP_3_ production by BRET, 3.6 × 10^5^ cells/well were transfected with 0.7 µg Luc-Akt-PH and 0.3 µg pYFP-Mem cDNAs^16,30^.

### BRET experiments

Twenty-four hours after transfection, CaSki cells were transferred into 96-well plates previously coated with polylysine (Perkin Elmer) and cultured for an additional 24 h in 1% FBS at 37 °C under 5% CO_2_ atmosphere, in the absence or presence of *O*-GlcNAcylation-inducing agents (10 µM Thiamet G (TG) + 5 mM GlcN). BRET experiments were performed as described previously^63^. In brief, cells were pre-incubated with coelenterazine (5 µM) during 10 min, and then stimulated with IGF-1 (5 nM). Light emission acquisition at 485 nm and 530 nm was then started immediately, and the BRET signal could be monitored during at least 20 min after IGF-1 addition. BRET signal was expressed in milliBRET units (mBU). The BRET unit has been defined previously as the ratio 530 nm/485 nm obtained when both luciferase and YFP were expressed corrected by the ratio 530 nm/485 nm obtained under the same experimental conditions in cells expressing only luciferase^64^. Each measurement corresponded to the signal emitted by the whole population of cells present in a well^65,66^.

### Western blot

CaSki cells were cultured in 6-well-plates for 24 h in 1% FBS, in the absence or presence of *O*-GlcNAcylation-inducing agents (10 µM TG + 5 mM GlcN) and then stimulated with IGF-1 (5 nM for 10 min). Cells were then washed with ice-cold PBS and lysed with buffer containing 50 mM Tris–HCl (pH 8), 137 mM NaCl, 10% (v/v) glycerol, 1% (v/v) Triton, 50 mM NaF, 10 mM disodium β-glycerophosphate, 1 mM Na_3_VO_4_, and protease inhibitors (1 µg/ml pepstatin, antipain, leupeptin, aprotinin, and AEBSF) supplemented with 10 µM PUGNAc to preserve the *O*-GlcNAcylation state of proteins during the extraction procedure^22^. Proteins (30 µg) were separated on 10% SDS-PAGE^67,68^, and then transferred onto nitrocellulose membranes (GE Healthcare), and after incubation with appropriate antibodies, the bands were visualized using enhanced chemiluminescence reagents (Thermo Fisher Scientific) with a FUSION FX7-Vilber Lourmat camera. The signals were quantified using ImageJ software.

### Immunoprecipitation of IGF1R

CaSki cells were lysed as described previously, and 300 µg of proteins were incubated with 1.5 µg of anti-IGF-1R antibody for 2 h at 4 °C. Immune complexes were collected by incubation with protein G-sepharose during 60 min at 4 °C, and washed three times with lysis buffer. The precipitated proteins were eluted in Laemmli buffer and analysed by SDS-PAGE followed by Western blotting.

### PTP1B activity assay

CaSki cells were lysed as described previously but using lysis buffer without Na_3_VO_4_. 300 µg of proteins were incubated with 1.5 µg of anti-PTP1B antibody (Calbiochem) for 2 h at 4 °C. Immune complexes were collected by incubation with protein G-sepharose beads during 60 min at 4 °C, and washed three times, first with lysis buffer and then with PBS. The activity of PTP1B was measured by incubating immune complexes immobilized on protein G-sepharose beads with pNPP buffer (24 mM HEPES pH 7.4, 120 mM NaCl, 100 mM pNPP, 5 mM DTT) at 37 °C during 90 min. The reaction was stopped by adding NaOH 30%. The beads were then centrifuged 2 min at 3000 rpm. The supernatant was removed and transferred into 96-well plates to measure the absorbance at 405 nm^69^.

### Cell growth (MTT) assay

CaSki cells were seeded in 96-well-plates at a density of 1.5 × 10^4^ cells/well and grown for 24 h at 37 °C in 5% CO_2_. Cells were then cultured in 1% serum in the absence or presence of 10 µM TG + 5 mM GlcN and 5 nM IGF-1. After 24 or 48 h of culture, cells were incubated with MTT (0.5 mg/mL) for 3 h at 37 °C. The reaction was stopped by adding acid isopropanol to each well. Formazan salts were dissolved and quantified at 570 nm using a microplate photometer (Multiskan FC, Thermo Fisher)^70^.

### Wound healing assay

Cells were seeded in 24-well-plates at density of 2 × 10^5^ cells/well and grown for 24 h at 37 °C in 5% CO_2_. After 24 h, a scratch was generated in the confluent monolayer using a sterile micropipette tip. Cells were rinsed with PBS and the wound margins were photographed (time 0) using an inverted microscope (Vert. A1 Zeiss) at 10 × magnification. Cells were then cultured in 1% serum in the absence or presence of 10 µM TG + 5 mM GlcN and 5 nM IGF-1. The wound margins were photographed 24 h and 48 h after the scratch^71^. Wound areas were quantified using Image J.

### Apoptosis analysis

CaSki cells were seeded in 6-well-plates at a density of 3 × 10^5^ cells/well and grown during 24 h. Cells were then cultured in 1% serum in the absence or presence of 10 µM TG + 5 mM GlcN, 5 nM IGF-1 and Cisplatin (5 µM) for 24 h. CaSki cells were then collected and rinsed in PBS. Apoptosis was measured using the Annexin-V-FLUOS Staining kit (Roche Diagnostics) according to the manufacturer’s instructions. Cells were analysed by flow cytometry (BD FACS Calibur cytometer). Data were analysed by FlowJo X software^71,72^.

### Immunofluorescence

CaSki cells were grown on a Chamber Slide System (Thermo Fisher Scientific) for 24 h in 1% FBS in the absence or presence of *O*-GlcNAcylation-inducing agents (10 µM TG + 5 mM GlcN) and then stimulated with 5 nM IGF-1 during 10 min. Cells were then washed with Tris-buffered saline (TBS) and fixed with 4% paraformaldehyde for 20 min at room temperature, and then washed with TBS. Non-specific binding sites were blocked with IgG-free 2% bovine serum albumin (BSA, Thermo Fisher Scientific) for 30 min at room temperature. Slides were then permeabilized for 10 min with 0.2% Triton X-100 in TBS and washed with TBS. The slides were incubated with Avidin solution for 10 min at room temperature, washed and incubated with Biotin solution for 10 min at room temperature (Biotin Blocking System, Dako). Slides were washed and then incubated with primary antibodies (anti-*O-*GlcNAc or anti-P-Akt) overnight at 4ºC in a humidity chamber. Slides were washed with TBS and incubated with secondary antibodies (FITC-conjugated anti-mouse and biotinylated anti-rabbit) at room temperature for 60 min in a dark humidity-chamber. Slides were then washed and incubated with streptavidin Alexa Fluor 594 for 30 min at room temperature. Slides were washed, stained, and sealed with Fluoroshield with DAPI-mounting medium (Sigma-Aldrich), and coverslipped^73^.

### Human tissues

All experiments were performed in accordance with relevant guidelines and regulations**.**

Studies on human biopsies have been approved by the Bioethics Committee of the Faculty of Medicine and Surgery of the University of Oaxaca (Universidad Autónoma Benito Juárez de Oaxaca, Mexico). Human cervical tissues were obtained from the Departamento de Patología del Instituto Nacional de la Nutrición y Ciencias Médicas “Salvador Zubirán”, México tissue bank. Informed consent was obtained from all participants. Biopsies were derived from patients with a diagnosis of invasive squamous cell carcinoma (n = 5) in clinical stage T1B1/FIGOIB1, according to the American Joint Committee Cancer/International Federation of Gynecology and Obstetrics system. Normal cervical tissues (n = 5) without inflammatory and infectious processes were derived from tissue remaining from hysterectomy for non-neoplastic indications.

The tissues were fixed in 4% phosphate-buffered formaldehyde and embedded in paraffin, the paraffin Sects. (3 mm) were deparaffinised, rehydrated and rinsed with Tris-buffered saline (TBS). Subsequently, slides were processed exactly as described previously^73^.

### Immunohistochemistry

Slides were observed through a Leica DM 2000 fluorescent microscope at 40 × objective. Images captured were analysed in Leica Application Suite Advanced Fluorescence 3.1.0 and Image J software.

### Hematoxylin and Eosin staining (HE)

For hematoxylin and eosin imaging, tissues stained with hematoxylin–eosin were observed with Primo Star Zeiss microscope at 10x, and photographed using Zeiss Axiocam Erc Rev 2.0 camera with ZEN 2.3 lite software.

### Statistical analysis

Statistical analysis were performed with GraphPad Prism software using Student’s t test for two groups comparisons, one-way-ANOVA followed by Tukey’s post-test for multiple comparisons, and Pearson’s test for correlations analysis.

## Supplementary Information

Supplementary Figures.
