# Supplementary material for: Increased O-GlcNAcylation promotes IGF-1 receptor/PhosphatidyI Inositol-3 kinase/Akt pathway in cervical cancer cells
Source: Sci Rep. 2022 Mar 16;12:4464. doi: 10.1038/s41598-022-08445-0 (PMC8927345; doi:10.1038/s41598-022-08445-0)

Supplementary Information

Increased *O*-GlcNAcylation promotes IGF-1 Receptor/Phosphatidyl Inositol-3 kinase/Akt pathway in cervical cancer cells

Victoria Jiménez-Castillo[1,2], Daniela Illescas-Barbosa[2], Edgar Zenteno[3], Beatriz Xóchitl Ávila-Curiel[2], Maria Cristina Castañeda-Patlán[3], Martha Robles-Flores[3], Daniel Montante-Montes De Oca[4], Eduardo Pérez-Campos [1], Anayetzin Torres-Rivera[6], Abdelouhab Bouaboud[5], Patrick Pagesy[5],Carlos Josué Solórzano-Mata[2]*,Tarik Issad[5]*.

[1] *National Technology of Mexico/IT.Oaxaca, Mexico*

[2] *Faculty of Medicine and Surgery/Faculty of Dentistry, Universidad Autónoma Benito Juárez de Oaxaca, Oaxaca, México*

[3] *Departamento de Bioquímica, Facultad de Medicina, Universidad Nacional Autónoma de México (UNAM), Mexico City, Mexico.*

[4] *Instituto Nacional de Ciencias Médicas y Nutrición Salvador Zubirán, Mexico city, Mexico*

[5] *Université Paris Cité, Institut Cochin, CNRS, INSERM, F-75014 Paris, France*

[6] *Tecnológico de Estudios Superiores de Huixquilucan, Mexico*

* Corresponding authors:
CJSM: universidad99@cecad-uabjo.mx
TI: tarik.issad@inserm.fr

CJSM and TI share senior co-authorship

Authors E-mails:
VJC: jimcv_28@hotmail.com
DIB: tomy_dib@hotmail.com
EZ: ezenteno@unam.mx
BXAC: beatrizavilacu@gmail.com
MCCP: cristi_ccp@bq.unam.mx
MRF: rmartha@unam.mx
DMM: danielmontantem@yahoo.com.mx
EPC: perezcampos@prodigy.net.mx
ATR: genyetzin@yahoo.com.mx
AB: abdelouhab.bouaboud@inserm.fr
PP: patrick.pagesy@inserm.fr

Supplementary figure legends

Supplementary Figure S1

O-GlcNAcylation-inducing treatments do not significantly affect IGF-1-induced Erk phosphorylation. CaSki cells were cultured in presence of 1% FBS, preincubated for 24 h in the absence or presence of TG (10µM for 24h) and GlcN (5 mM for 6h). Cells were then stimulated with IGF-1 (5nM) during 10 min. Erk phosphorylation levels were analysed by western-blotting using the indicated antibodies. Histograms represent the means ± SEM of the ratios of P-Erk/Erk signals in 6 independent experiments. Statistical analysis was performed using ANOVA followed by Tukey's post-test. *, **: $p<0.05$ and $p<0.01$, respectively. NS: non-significant.

Supplementary Figure S2

Separated effects of Thiamet-G and glucosamine in CaSki cells

(A) Effect of Thiamet G alone and glucosamine alone on protein O-GlcNAcylation profile in Caski cells. Cells were cultured in 1% FBS and cultured in the absence or presence of TG (10 µM) for 24 h, GlcN (5 mM) for 6 h and/or IGF-1 (5 nM) for 10 min. Cell lysates were collected and analyzed by western blot with anti-*O*-GlcNAc antibody. Histograms represent the means ± SEM of the ratios of O-GlcNAc to GAPDH signals in 2 independent experiments.

(B) Effect of Thiamet G alone and glucosamine alone on basal and IGF1-induced PIP3 production in CaSki cells. CaSki cells were co-transfected with cDNAs coding for the PH domain of Akt fused to a luciferase (Luc-Akt-PH) and a plasma membrane-targeted YFP (YFP-mem). 24h after transfection, cells were cultured in the presence of 1% FBS in the absence or presence of TG (10µM for 24h, upper panel) or GlcN (5 mM for 6h, lower panel). Cells were incubated with coelenterazine for 10 min, and then stimulated with IGF-1 (5nM). Light emission acquisition at 480 nm and 532 nm was started immediately after IGF-1 addition. Results were expressed in miliBRET units (mBU). Left panel: a typical real-time experiment showing the effect of *O*-GlcNAcylation-inducing treatment on IGF-1-induced PIP_3 production in CaSki cells. Right panel: Results are expressed as the delta BRET (increased BRET above basal) and are the means ± SEM of 6 independent experiments.

(C) Effect of Thiamet G alone and glucosamine alone on basal and IGF1-induced cell growth. Cells were cultured in 1% FBS and cultured in the absence and presence of TG (10 µM, left panel) or GlcN (5 mM, right panel) and/or IGF-1 (5 nM). MTT assay was used to determine the cell growth at 24h and 48h. Results are the mean ± SEM of 5 independent experiments.

Supplementary Figure S3

Effect of Thiamet-G and glucosamine on protein N-glycosylation in CaSki cells

(A) CaSki cells were cultured in 1% FBS in the absence and presence of TG (10 µM, 24 h) GlcN (5 mM, 6 h) or both. Cell lysates were submitted to SDS-PAGE followed by western-blotting with biotinylated lectins (ConA, PHA-L and WGA). The blots were revealed using ExtrAvidin-Peroxidase. Results are mean ± SEM of 3 independent experiments.

(B) CaSki cells were cultured in 1% FBS in the absence and presence of TG (10 µM, 24 h) GlcN (5 mM, 6 h) or both. Cells were fixed, incubated with biotinilated lectins, washed and incubated with Alexa Fluor-594 streptavidin and then analysed by FACS. Results are the mean ± SEM of 2 independent experiments.

Supplementary Figure S4

Effect of O-GlcNAcylation-inducing treatments on IGF1R/PTP1B interaction and on PTP1B O-GlcNAcylation and tyrosine phosphorylation status

(A) Effect of TG+GlcN on basal and IGF1-induced interaction between IGF1R and PTP1B in CaSki cells. CaSki cells were co-transfected with cDNAs coding for IGF1R fused to a luciferase (IGF1R-Luc) and a YFP-tagged substrate-trapping mutant of PTP1B (YFP-PTP1B-D181A). 24h after transfection, cells were cultured in the presence of 1% FBS in the absence or presence of TG (10µM for 24h) and GlcN (5 mM for 6h). Cells were incubated with coelenterazine for 10 min, and then stimulated with IGF-1 (5nM). Light emission acquisition at 480 nm and 532 nm was started immediately after IGF-1 addition. Results were expressed in miliBRET units (mBU). Left panel: a typical real-time experiment showing the effect of treatments on IGFR/PTP1B interaction in CaSki cells. Right panel: Results are expressed as area under the curve (AUC, mBU.min) and are the means ± SEM of 5 independent experiments.

(B) CaSki cells were co-transfected with cDNAs coding for IGF1R and a YFP-tagged substrate-trapping mutant of PTP1B (YFP-PTP1B-D181A). 24h after transfection, cells were cultured in the presence of 1% FBS in the absence or presence of TG (10µM for 24h) and GlcN (5 mM for 6h). Cells were stimulated with 5nM IGF1 for 10 min and then lysed at 4°C in buffer containing protease, tyrosine-phosphatase and O-GlcNAcase inhibitors as described in the method section. YFP-PTP1B was then immunoprecipitated using anti-GFP antibody (Roche), submitted to western-blotting and revealed with either anti-OGlcNAc antibody (RL2). Blots were then reprobed with anti-PTP1B antibody. PTP1B does not appear to be O-GlcNAcylated upon treatment with TG+GlcN, whereas a marked increase in protein O-GlcNAcylation was observed

in total cell lysates under these conditions. Immunoblots are representative of 4 independent experiments.

(C) YFP-PTP1B was immunoprecipitated using anti-GFP antibody, submitted to western-blotting and revealed with anti-phosphotyrosine antibody (4G10). Blots were then reprobed with anti-PTP1B antibody. Immunoblots are representative of 4 independent experiments. Histograms represent the means ± SEM of the ratios of pY-PTP1B to total PTP1B signals in 4 independent experiments.

Supplementary Figure S1

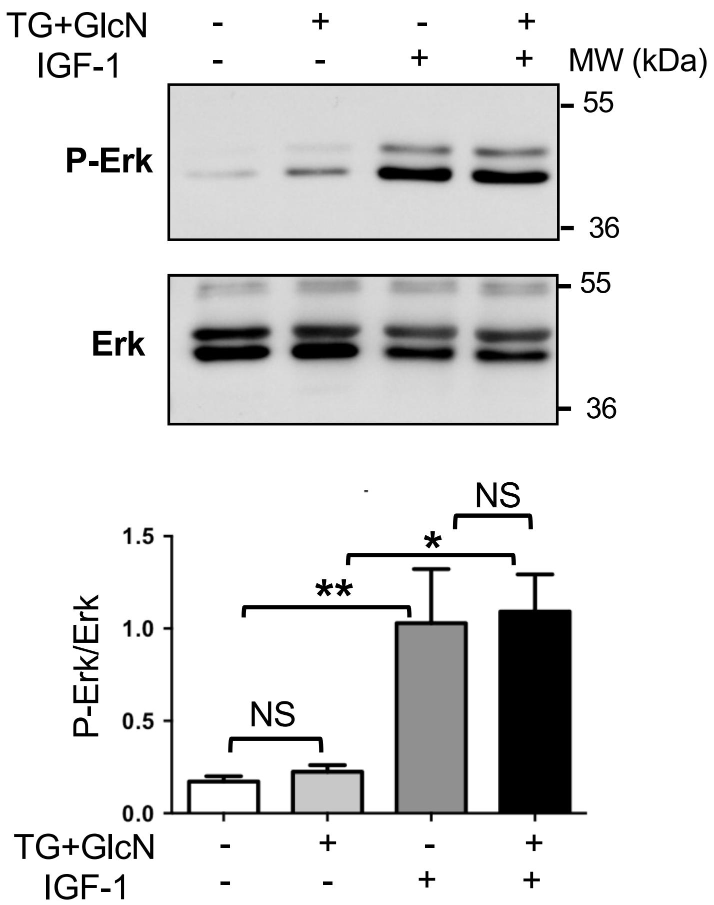

Supplementary Figure S2

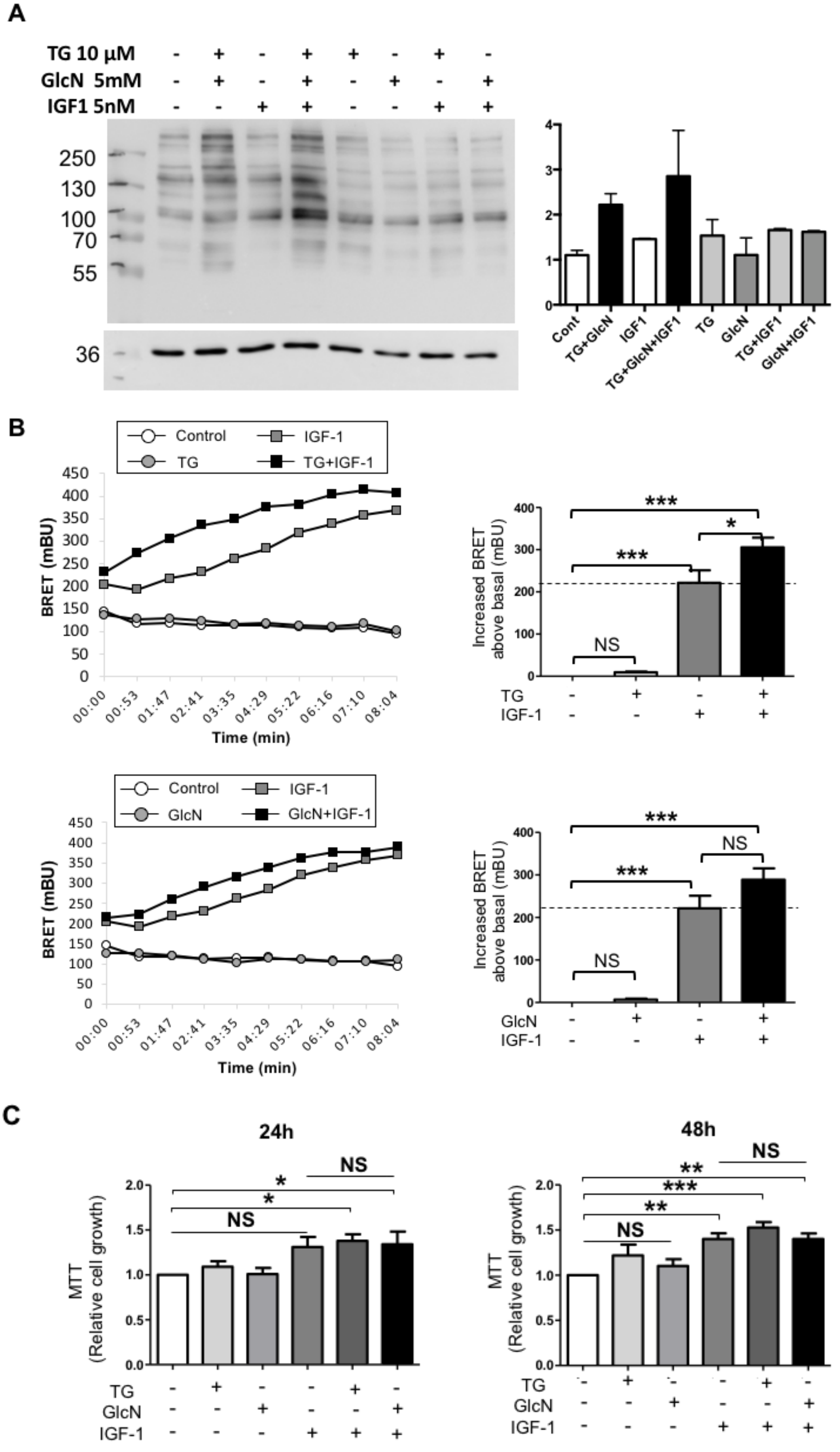

Supplementary Figure S3

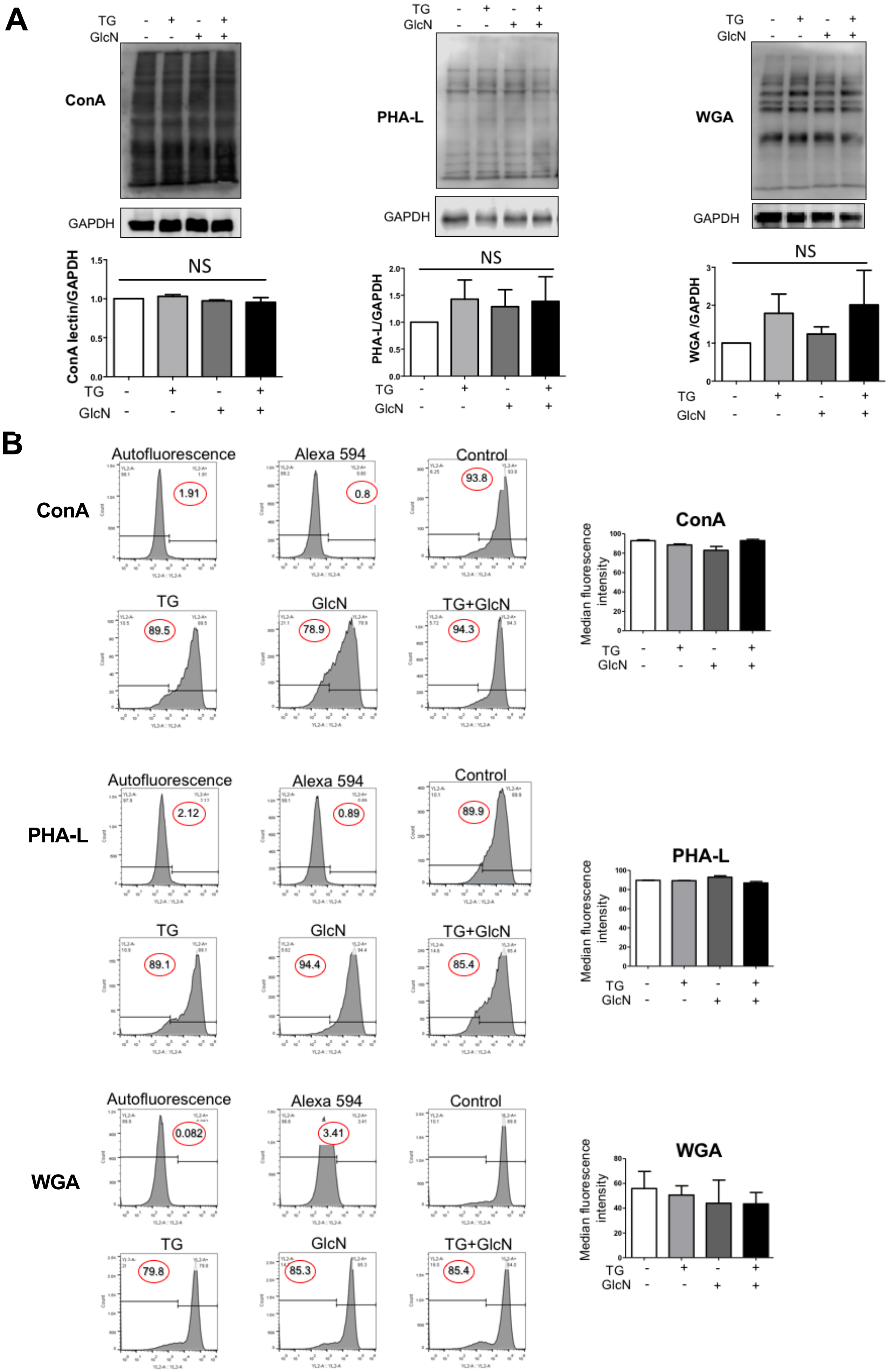

Supplementary Figure S4

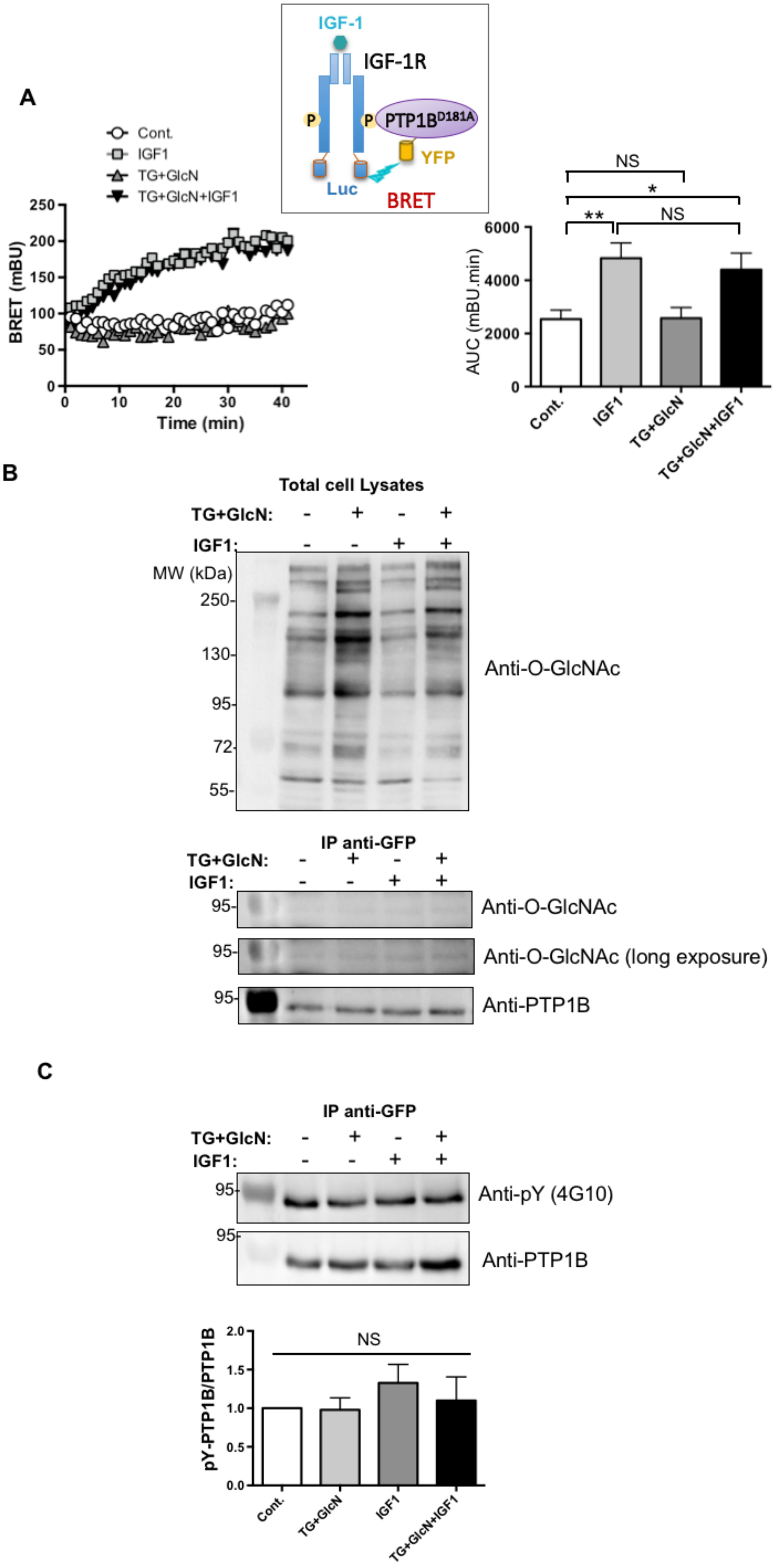

Supplement: Supplementary file 1 — Supplementary Figures. [file 41598_2022_8445_MOESM1_ESM.pdf]
